# Types of Adolescent Screen Use and Positive Wellbeing: Gender and Parental Education Influences

**DOI:** 10.1007/s10902-025-00884-6

**Published:** 2025-04-12

**Authors:** Grace Chang

**Affiliations:** Department of Sociology, University of Oxford, 42 Park End St, Oxford, OX1 1JD UK

**Keywords:** Youth, Screen use, Media, Happiness, Flourishing

## Abstract

Research has contradictory findings because of different definitions of screen time, measures of wellbeing, and the examination of different groups of teenagers. This study distinguishes four types of screen activity using time diaries of UK adolescents: social screen time, internet browsing, playing e-games and video viewing, and examines their associations with adolescents’ happiness in six domains, by gender and parental education. Any form of screen time is associated with lower happiness with looks, but worst for the former two activities. These activities are also associated with lower happiness in other domains, and excessive use equate to worse wellbeing. More screen time is worse for girls’ happiness with their looks than boys, and parental education is not a protective factor.

## Introduction and Background

There has been a rapid rise in digital device use among children and adolescents in the last two decades. Internet use among 12 to 15 year olds in the United Kingdom (UK) more than doubled from 8 h a week in 2005 to 18.6 h a week in 2015, with mobile phone and tablets being more popular devices for internet access than computers (Ofcom, 2015). Adolescence is a period of dynamic brain development and exposure to stimuli, which is important for adolescents’ skills acquisition and emotional processing (Dahl et al., 2018). Coupled with the fact that adolescents are also early adopters of new technologies (Dahl et al., 2018), there is heightened parental and societal concern about screen use and adolescents’ wellbeing, as highlighted by the recent UK House of Commons Education Committee report (House of Commons, 2024).

Despite growing research examining the relationship between screen use and wellbeing, studies report mixed findings because of the use of different definitions of screen time and measures of wellbeing, and different groups and characteristics of teenagers (Orben, 2020; Stiglic & Viner, 2019; Hoare et al., 2016, Dickson et al., 2018, and Best et al., 2014). In addition, studies have shown that girls are more vulnerable to risky screen content such as body image pressures because girls undergo puberty earlier than boys (Dahl et al., 2018), and are exposed to early sexualised ideation of females on media (APA, 2007). However, fewer studies examine the interaction of both gender and parental education. For instance, whether children with higher parental education may have stricter parenting styles around media, or use diverse ways to mediate screen use such as going to museums (Livingstone et al., 2015).

This study examines three research questions: One, how do four different types of screen use relate to adolescent’s positive wellbeing? Two, does excessive screen time equate to worse wellbeing? Three, how do these relationships vary by gender and parental education? To do so, I distinguish four different types of screen use typically cited in the literature but not distinguished in one analysis; (1) social screen time (e.g., social networking sites or texting/emailing); (2) internet browsing; (3) playing video games or apps (from here on referred to as electronic games or e-games) and; (4) passive video viewing. Using time diaries of 14-year-olds born in 2000/02 from the UK Millennium Cohort Study (MCS) in 2015, I examine how screen use is associated with adolescents’ happiness in six domains, using lagged dependent value models, controlling for previous wellbeing and extensive background characteristics.

This study contributes to the literature by documenting screen time comprehensively, examining its’ association with several domains of adolescent happiness, where previous research has focused on mental health disorders. It also adds to our knowledge about the variations of these relationships by *both* gender and parental education, of which little is known about.

## Prior Literature and Background

### Internet Browsing and Social Networking Sites (SNS) and Wellbeing

Internet browsing and SNS use involve greater social interactions with other people than watching videos or playing games. Adolescents may gain opportunities for improved mental wellbeing through increased social support, widening social networks and connections, and accessing global information (Best et al., 2014; Livingstone et al., 2018). On the flipside, adolescents are at risk of being exposed to harmful content such as cyber-bullying or peer pressure (Best et al., 2014). For UK adolescents aged 10 to 15, Mcdool et al. (2020) finds that time spent online was inversely related to their happiness, with negative associations stronger for girls than boys, and the largest effect being associated with unhappiness with their appearance. However, they focused on internet use more broadly, and they find that a key mechanism in the relationship between internet use and wellbeing was from excessive use of SNS.

SNS is a more mobile, immersive, and continuous form of screen time (see Orben (2020). They tend to be image-based platforms which allow high frequencies of image sharing and quick image-manipulation techniques. This has led to concerns about promoting an ‘idealised’ body image or lifestyle, negatively associated with self-image. Studies using retrospective screen time measures mainly document a negative relationship between social media and adolescent wellbeing (Booker et al., 2018; McNamee et al., 2021) and a higher risk of depressive symptoms (Kelly et al., 2019), especially for girls compared to boys (Booker et al., 2018; Kelly et al., 2019; McNamee et al., 2021). Girls may be more vulnerable because they tend to be exposed to early sexualisation through peer pressure, and the depiction of females in pop culture and the general media (APA). In contrast, some studies find no significant, or weak associations between social media and mental wellbeing (Coyne et al., 2020; Leung, 2014; Puukko et al., 2020) or are very small (Orben, 2020).

Few studies use time diaries, and two studies that include the MCS time diary data of 13 to 15 year olds reported opposite findings. Banthorpe et al. (2020) finds that more social media use was associated with a greater probability of self-harm and depression and lower self-esteem, especially for girls. However, Orben and Przybylski (2019), using time diaries from Ireland, the US and the UK, find little evidence of a negative association between digital-screen engagement and adolescent wellbeing. These differences may be due to the screen use measure; Orben and Przybylski (2019) use a measure of total screen time, while Banthorpe et al. (2020) only examines social media use.

Studies in the UK and the USA also show that greater internet browsing and SNS use crowd out time for activities that promote mental wellbeing such as sleep (Hisler et al., 2020), and face-to-face interactions (Twenge et al., 2019). Hisler et al. (2020) found that SNS and internet use were more strongly associated with shortened sleep duration than gaming or TV use, possibly because they involve more immersive social interaction with other people, or are typically carried out on portable devices held closer to the face before sleep. In contrast, Orben and Przybylski (2020) re-examined this relationship using time use diaries for MCS and found that screen use before bedtime was not substantively associated with hours of sleep. Zhang et al. (2022) using 2017 data of Italian adolescents found that problematic SNS use is associated with lower happiness because of greater difficulties in falling asleep and lower frequencies of physical exercise.

## Playing e-games, Watching Videos, and Wellbeing

Concerns about gaming and video viewing (watching TV) have some overlaps. Studies have found links between excessive time spent on TV and gaming, such as being exposed to violent content, and exhibiting greater aggressive behaviour, and worse prosocial behaviour (Anderson et al., 2010). However, gaming is distinct from watching videos in that it requires active engagement with the game, usually involving some level of motor function. It may reward communication and cooperation, as well as resolving negative emotions and frustration (Granic et al., 2014). While evidence on the positive link between gaming and wellbeing is lacking, some studies have found no statistically significant associations between violent and non-violent gaming and adolescent aggression (Ferguson, 2015; Przybylski & Weinstein, 2019). An additional concern about watching TV is that studies suppose that excessive time watching TV leads to higher amounts of sedentary time (Hoare et al., 2016), and/or less healthy diets (Stiglic & Viner, 2019), which is associated with poorer mental health (Kandola et al., 2020). While many screen activities such as internet browsing and playing games are linked to more sedentary behaviour, the majority of this evidence is for watching TV (Hoare et al., 2016).

All of the findings described above assume a more-is-worse relationship with each of the screen activities, which may not necessarily hold. In contrast, the “Goldilocks Hypothesis”, which was first coined by Przybylski and Weinstein (2017), posits that there may be a level of screen time that is ‘just right’. The authors argue that ‘too little’ screen time reflects adolescent deprivation of social information whereas ‘too much’ may lead to displacing beneficial activities. They show that for English 15-year-olds, moderate amounts of self-reported screen time are associated with higher mental wellbeing compared to very low or very high levels of screen time. However, this hypothesis is controversial. Research using self-reported screen time shows support for it (McNamee et al., 2021), but studies that use time diaries do not (Sanders et al., 2019).

## Variations by Gender and Parental Education

As discussed above, girls usually face greater stresses and pressures from SNS and the internet, but this can also be present in playing e-games and watching videos. Although studies find that boys spend more time on digital activities than girls (Becker, 2022; Gracia et al., 2022), the online risk for girls are much greater (Hartas, 2019). This may also be driven by how the distinct type of screen activity is used. For instance, a study of adolescents aged 13 to 17 in the USA in 2015 by Lenhart (2015a) show that boys use e-gaming as a platform for friendships while girls use social media and texting, which is more challenging (e.g., girls are more likely to block friends). Jürges and Khanam (2021) find that although boys spend more time in front of screens, more screen time instead of physical activity has a greater negative impact on girls’ social and emotional competencies than boys.

Studies also discuss the variations of screen use by socio-economic status, usually proxied by parental education or family income. The literature discusses three ’levels’ of digital divides across the population; (1) the unequal access to screens or information and communications technology (ICT), (2) unequal online and digital ’skills’ and divergent forms of engagement, and (3) multiple outcomes that stem from dissimilar social and digital contexts (Hargittai, 2008; Helsper, 2021). To date, there is little systematic analysis of how changes in digital engagement relates to wellbeing across socio-economic groups, especially with regards to levels two and three (Gracia et al., 2023). Studies find that adolescents from lower socio-economic backgrounds spend higher amounts of time on screen-based activities, experience more negative feelings during their online activities, and have scarcer economic, social, cultural and digital resources to help secure a healthy and productive engagement with screens (Gracia et al., 2023; Helsper, 2021).

However, adolescents from higher socio-economic backgrounds may also be exposed to more risk of harmful content because of better access to the internet and digital technologies (Livingstone et al., 2015). The authors find that children from higher socio-economic homes compared to lower ones, are more likely to see online sexual images, and to receive more sexual messages online, but this exposure may not relate to subjective harm, possibly mitigated by parenting (Livingstone et al., 2011). Conversely, Lenhart (2015b) show that lower income teenagers are more likely to have the same friends over multiple social media platforms, which may indicate greater selection of friends because they have limited access to these technologies. Recent studies also show that adolescents with higher socio-economic status use technology as frequently as others and in excess of recommendations, partly due to parents’ ambivalence about technology and perception that expert guidance is unrealistic (Mollborn et al., 2022). Thus, there may not be any differences across socio-economic groups.

There may also be an interaction effect between gender and parental education. Parental education may provide more protective factors for girls, where parents can understand how to mediate their screen time through other means (e.g., outdoor activities). On the flipside, we may also see a double-whammy of negative associations between screen time and girls with low parental education, shown in previous studies (Männikkö et al., 2020). Therefore, we may expect boys of high parental education to fare best, and girls of low parental education to fare worse, and the other two groups in between. On the other hand, if adolescents regardless of parental education use screen time equally irresponsibly (Mollborn et al., 2022), then we may not see any differences by parental background.

## Adolescents’ Screen time in the UK

Examining adolescents’ screen time in the UK in 2015 is relevant because children born in the millennium have grown up with screens, the internet, and SNS. Mullan (2019) documents that children aged 8 to 16 in the UK led less physically active, and more home-based lives in 2015 compared to children in 2000, driven by a substantial increase in screen-based activities. Ofcom (2015) reports that in 2015, 12- to 15-year-olds in the UK had three or more devices of their own, typically a smartphone, a tablet and a laptop/PC, with few differences by gender or household socio-economic status. The report also indicated that YouTube became an important source of content in addition to watching TV. 86% of 12- to 15-year-olds in 2015 who watched TV also watched YouTube. Of those who watched both types of content, for the first time in 2015, more adolescents preferred to watch YouTube videos compared to TV channels. Popular SNS platforms used at this time were Facebook, Instagram, Snapchat and YouTube.

There are strong parental and societal concerns about adolescents’ wellbeing and screen use in the UK. Ofcom (2015) reported that one-third of parents whose child goes online were concerned about online bullying and about a fifth to a quarter of parents were concerned about content viewed on TV and the internet, respectively. These concerns are also highlighted at the policy level, as reflected in reports such as by the House of Commons (2024), which reviewed studies on the impact of SNS and screen use on young people’s health. In addition, understanding these relationships for adolescents in 2015 helps uncover conservative estimates for us to understand the potential relationships since Covid-19.

## Screen time since 2015

Adolescents’ overall screen use has risen rapidly since 2015, especially during the pandemic, with little evidence that pre-pandemic screen levels have returned as technology becomes integrated into our daily lives (Lua et al., 2023). During the pandemic, time on social media and the internet in particular rose (Madigan et al., 2022), but evidence about its implications on wellbeing is mixed. During the pandemic lockdowns, studies have shown that screen time may be beneficial in promoting greater physical activity (gamification of exercise indoors) and maintaining social connectedness, but may have been particularly detrimental for sleep outcomes (Lua et al., 2023). However, these findings relate to a very special time, where there were few alternatives to being at home during lockdown.

Adolescents today engage with social media and the internet in new ways, thus how they behave on these platforms are important. Winstone et al. (2022) show that adolescents who are “Broadcaster” users frequently share content in addition to socialising or browsing online – are at greatest risk to their mental wellbeing, compared to other groups which frequently use messaging and browsing, but lower content sharing. Adolescents with greater resources may be more able to ‘broadcast’ and share content compared to adolescents who have lower resources. Adolescents with higher educated parents are also likely to be friends with other adolescents that have families with resources, which may fuel a competition for upward comparisons through internet platforms. While I do not observe the types of content/platforms used, these differences may explain the channels in which there may be differences across demographic groups.

Although there are differences in screen use since 2015, the concerns about adolescent screen use today are still similar. The evidence about the harms or benefits of screen use still depends on various contexts such as how the platform is used, which screen activity (and within each type of screen, which platform), and how it is defined, and the overall time use patterns of adolescents including both online and offline activities (Madigan et al., 2022; Lua et al., 2023). Therefore, this research aims to elicit the associations between adolescent happiness and screen use in comparison to other activities within a 24-hour day in a context where screen time is used differently to today, but acknowledges that it provides lower bound estimates to understand future relationships, while social surveys collect new information about adolescent behaviour with screen use.

### Data, Measures and Methods

#### The Millennium Cohort Study

The MCS is a nationally representative, longitudinal birth cohort survey of individuals born in England and Wales between 2000/02 (UCL, 2023). The MCS data has rich information about adolescents, parents, and teachers over eight sweeps (between the average age of 9 months and 23 years old). The sample covered children from England, Wales, Scotland and Northern Ireland in the UK who were eligible for child benefits and were 9 months old at the time of the first sweep. It used a stratified, clustered random sample design and oversampled from areas that were disadvantaged or had high ethnic minority populations. My analysis uses time use diary (TUD) data from the sixth sweep of the MCS data in 2015, when the adolescent was 14-years-old. The MCS had an initial sample of 18,818 children who were surveyed in sweep one; this figure had fallen by 60% to 11,884 adolescents in sweep six. I use non-response weights for sweep 6 to account for attrition (Ketende & Jones, 2011).

Only 10,337 adolescents were invited to complete the TUD because of limited activity monitoring devices (administered alongside the TUD to measure physical activity), comprising all adolescents in Wales, Scotland, and Northern Ireland and approximately 81% in England. Of the eligible sample, 4,640 adolescents completed and returned the TUD. 3,919 answered both the weekend and weekday diaries, 353 only the weekday and 368 only the weekend. This corresponds to approximately 24% of the original sample in sweep one. Appendix Table A1 shows the sample differences for adolescents who have matched TUD compared to those who did not match. The matched TUD samples showed similar representation by age, but is biased towards girls, white ethnic groups, and adolescents from Wales, Scotland and Northern Ireland. However, since my analysis uses a cohort study about screen use in 2015, the analysis with or without the response bias from TUD information, do not generalise to other adolescents in the UK.

When comparing TUD information to other variables of interest, most missing information is from adolescents’ happiness scores at age 11. Since I cannot determine whether the missing information is due to actually not knowing the answer or a refusal, I restrict my sample to all non-missing relevant information, which leads to a loss of about 28% of the original sample, resulting in a final sample of 3,340 weekday time diaries and 3,370 weekend time diaries. While the randomization of the sample issued can at least represent the sample by country, the final sample is selected and non-random. There is likely non-response bias on returned TUD, as the TUD is likely more cumbersome than the standard questionnaire, and on top of that, significant self-selection for cohort members that continue in a longitudinal study.

The TUD provides extensive information about the adolescents’ primary activities in two 24-hour periods, a randomly chosen weekend and weekday, recorded using one of three possible modes: an app, paper, or online. The smallest activity time slot was 1-minute for the app, and 10-minute slots online or on paper. The adolescents were required to provide full details of what they did between 4am one day and 4am the next day, choosing from a pre-coded list of 44 activity codes, nested within 12 top-level activity categories. Table 1 and Appendix Table A2 show the full list of activity codes and grouped activities. The regular public-use MCS provides a harmonised version of the data that standardises all responses from the three modes to 10-minute slots, but omits information such as who they performed the activity with. I added value to these data using previously unprocessed information, matching information on who the activity was done with to the harmonized data using the code provided in the MCS technical report (Veeravalli, 2019).

## Measures of Screen Time, Happiness, and Covariates

The TUD provides a list of six pre-coded distinct screen activities, which I re-categorised into four types; social screen time (three of the pre-coded activities merged into one), internet browsing, playing e-games, and video viewing. The categories are summarised in Table 1 below. As discussed in the literature review, previous research often discuss the implications of internet browsing and social networking sites as one screen activity, in comparison to playing e-games and watching videos as another activity, as the former is more concerned with social comparisons due to interaction with peers, and the latter is more concerned about sedentary behaviour and aggression.

For each of these major groups, I further distinguish between more ‘active’ and ‘passive’ activities. That is, social screen time and playing e-games require more active engagement with peers or a game, while internet browsing and watching videos are more ‘passive’ in absorbing readily available content. Since the data is in 2015, popular platforms that allowed interaction with peers online included Facebook (social networking sites) and Skype (video calls). Texting and messaging were also still prevalent forms of connectivity, where WhatsApp gained its highest popularity in 2015 globally (Metz, 2016), and thus I grouped these activities as ‘social screen time’.

**Table 1:** Four types of screen activities from the MCS time diaries

Grouped activities	Pre-coded activities in the diary data
1. Social screen time	- Answering emails, instant messaging, texting - Speaking on the phone (incl. Skype, video calls) - Updating/browsing social networking sites
2. Internet browsing	- General internet browsing, programming (excl. social networking sites)
3. Playing e-games	- Playing electronic games and apps
4. Video viewing	- Watching TV, DVDs, and downloaded videos

Note: Each raw pre-coded activity is indicated by the bullet point in the second column

Of the remaining 37 activity codes, I distinguish five additional types of activities that may be displaced by screen time as discussed in the literature previously: sleep, physical activity and exercise, educational activities, work/leisure alone, and work/leisure with others. I also control for these activities in the regression estimates, as adolescents who spend a similar number of hours on screen time may spend different amounts of time on say, physical activity (see full list in Appendix Table A2).

My main outcome of interest is in the adolescent’s positive subjective wellbeing, which I measure using adolescent happiness ratings in six domains; the way they look, friends, family, school, school work, the school they go to, and their life as a whole. This measure was first conceptualised by Huebner (1994) to create a multidimensional measure of positive subjective wellbeing for children and adolescents. While happiness with the way they look is more strongly related to self-concept, happiness with friends, family, school and school work all reflect healthy peer engagement in environments that adolescents spend most of their time in (Zǔkauskiene, 2014). For each domain on the happiness scale, the responses range from “Not at all happy”=1 to “Very happy”=7. Each happiness domain score is standardised to have a mean of zero and a standard deviation of 1.

I use parental education as a proxy for socio-economic status, measured by a binary variable equal to 1 if the highest parental education in the household is above the level NVQ4 (equivalent to an undergraduate degree or a full technical certification), and 0 otherwise. I use parental education because parents with higher education not only have more assets and resources, but may also have different views, knowledge, and parenting styles around screen use (see Appendix Table A3 for the correlation between parental education, income, and social class).

I use several individual and family-level controls to account for observed differences between adolescents’ screen use and mental wellbeing. Since family characteristics have the greatest effect on adolescent’s later development (Francesconi & Heckman, 2016), I use the earliest measures of highest social class in the family and housing tenure as wealth proxies. To account for potential transmission of wellbeing through genetics, I control for the main parent’s mental health and the adolescent’s mood score when they were an infant. I also control for parenting with respect to media at age 11, the latest age this information is collected.

At the individual level, I control for age, ethnicity, the presence of any long-term illness; and their cognitive score measured by the word-activity score. I also control for family demographics, such as number of siblings, which may determine device sharing, mother’s age at the birth of the child, and whether both natural parents were in the household, to control for family hardship and parental income/time resources. I control for the disadvantaged and advantaged stratum indicator variables, and the number of days and mode of reported TUD.

Appendix Table A4 describes the background characteristics of the adolescents by parental education and gender. The groups are similar in terms of age, ethnicity, health, and parenting styles. Adolescents with lower parental education had slightly more siblings, had lower word scores, had younger mothers, were more likely to rent their home, more likely to not have a father at home, and more likely to have not working, semi-routine and routine jobs as the highest social class in their family, and slightly higher parental depressive scores. They are also slightly more likely to report one day in the TUD, and use the paper mode to complete the survey. Girls with high parental education had the best infant temperament score, except for regularity, where boys with low parental education had the best score.

### Modelling the Screen time and Wellbeing Relationship

I examine the relationship between adolescents’ screen time and their wellbeing, using the specification below. For each adolescent at the average age of 14, I estimate:

Y = α + β S + γI + δ G + εV + ζ T + ηX_5_ + θY_11_ + ιR + κ

Where Y represents one of the three mental wellbeing measures. The screen measures are indicated by social screen time (S), internet browsing (I), playing e-games (G), and video viewing (V), respectively. The specification controls for all adolescent, demographic, and parent characteristics as described (X_5_). T is the full vector of “other” time activities such as sleep, physical activity, and so on, with sleep as the base category in my main specifications. As discussed in Zhang et al. (2022), more screen time at the cost of physical activity or sleep are often the discussed mechanisms in explaining the relationship between screen time and wellbeing. Estimates using physical activity as the baseline is also included in the Appendix with similar estimates, and smaller magnitude (see Appendix Table A7 and A8). Y_11_ is the adolescent’s happiness measure at age 11, corresponding to the same happiness domain at age 14, Y. The coefficients β, γ, δ, and ε measure the association of an additional hour of the respective screen time and mental wellbeing, instead of the baseline activity in T, sleep. R is stratum indicator variable to control for regional differences and κ is the error term. I fit the model separately by weekday and weekend since activities are likely to differ by these days, especially with respect to screen use.

First, I examine whether an additional hour of each screen activity instead of sleep is associated with greater or poorer happiness, which refers to coefficients β, γ, δ, and ε. I then examine whether there is a rank order in size of the coefficients, and the magnitude of the coefficients by increasing hours of screen use. Then, I run the model separately for groups of teenagers by four groups; girls with high parental education, girls with low parental education, boys with high parental education, and boys with low parental education, to examine the heterogeneity in these relationships.

A concern when estimating the relationship between adolescents’ screen time and their wellbeing is reverse causality. For example, adolescents with lower happiness may also spend more time on SNS. A second concern is unobserved heterogeneity, i.e., that there are unobserved factors (e.g., peer pressure) that may affect both screen behaviour and happiness.

To partially account for these problems, I follow previous studies by including a lagged dependent variable (LDV) (Keele & Kelly, 2006; O’Neill et al., 2016). The LDV is a proxy to capture all previous observed or unobserved inputs (e.g., unobserved ability) experienced by the adolescent up to age 11. For example, if an adolescent’s parent has strict screen times for their child, who has a good aptitude for school, then the LDV proxies for these observed inputs (parents’ screen time rules) and unobservables (aptitude for school) because the inputs by the parents or adolescent are reflected in his/her lagged happiness score. In addition, I control for extensive observed individual, parental, and demographic characteristics. The main limitation of the LDV model is that it makes strict assumptions that the effects of all of the inputs on skills formation must decline at a constant rate, across age, i.e., the effect of all inputs at age 11 is greater than the effects of inputs at age 14 on happiness, which may not necessarily be true. Since my study examines adolescents’ happiness as a cumulative dynamic process, I follow Keele and Kelly (2006), who argue that omitting a lagged dependent variable (LDV) can bias estimates using ordinary least squares.

### Findings

#### 14-year-olds’ Screen time and Other Activities

Table 2 reports the proportion of adolescents engaged in each activity (column A), and the average hours spent on the activity for adolescents engaged in the activity (column B). The majority of adolescents engaged in any screen activity on a weekday (82%) and the weekend (87%), mostly watching videos, followed by social screen time, playing e-games and browsing the internet. Conditional on engaging in the activity, adolescents spent the most time playing e-games compared to other screen activities − 2 h 43 min on a weekday and 3 h 18 min at a weekend, followed by video viewing (2 to 3 h), social screen time and internet browsing (1 to 1 h 45 min).

There is a gender division in screen use. Boys were more likely than girls to play e-games (1 to 2 h more) while girls were more likely to engage in social screen time than boys (over half compared to a third), but hours spent on social screen time conditional on engagement were similar across groups, only slightly higher for girls with low parental education. Most group differences in screen time are evident on a weekday.

A higher proportion of girls and boys with high parental education engage in any form of screen activities, especially browsing the internet and watching videos, compared to those with lower parental education. However, adolescents with lower parental education spend more time on the screen activity, conditional on engagement. Overall, these gaps are larger between gender compared to parental education.

It is worth noting that there is unlikely a digital divide by age 14. In Appendix Figures A1 and A2, ownership and access to ICT at the household level are the same at age 14, across parental education. The only gap is that two thirds of children with lower parental education had a TV in their bedroom at ages 7 and 11 compared to a third of children with higher parental education. However, I do not observe direct ownership of devices by the adolescent, thus I cannot account for the quality of internet or exclusive ownership of devices for these adolescents, a limitation of the dataset.

**Table 2:** Average engagement and conditional engagement in activity by gender and parental education

	(A) Percentage engaged in activity, %						(B) Average hours conditional on engagement (SD)					
	All	*Girls*		*Boys*			All	*Girls*		*Boys*		
		High	Low	High	Low	p-value		High	Low	High	Low	p-value
**Weekday**												
All screen activities	82.27	84.70	78.27	86.31	80.99	**	3.54 (2.51)	2.73 (2.10)	3.05 (2.12)	4.05 (2.66)	4.49 (2.77)	***
Social screen time	45.13	57.74	53.42	33.51	33.10	***	1.56 (1.59)	1.34 (1.44)	1.71 (1.61)	1.54 (1.77)	1.64 (1.61)	*
Internet browsing	12.87	13.31	10.06	16.74	12.28	*	1.39 (1.46)	1.01 (1.04)	1.44 (1.32)	1.51 (1.44)	1.60 (1.89)	**
Playing e-games	28.90	13.07	8.79	51.81	48.23	***	2.72 (2.34)	1.24 (1.54)	1.52 (1.46)	2.82 (2.31)	3.29 (2.45)	***
Video viewing	54.32	60.49	52.02	55.94	49.45	**	2.30 (1.64)	2.06 (1.45)	2.30 (1.58)	2.26 (1.75)	2.64 (1.75)	***
Unweighted Obs	3340	970	874	858	638		2779	825	702	735	517	
**Weekend**												
All screen activities	86.95	88.47	85.72	88.39	85.61	*p* = 0.288	4.77 (3.11)	4.01 (2.54)	4.06 (2.74)	5.42 (3.31)	5.72 (3.39)	***
Social screen time	48.08	59.32	61.97	33.29	33.75	***	1.69 (1.74)	1.68 (1.69)	1.81 (1.74)	1.41 (1.70)	1.60 (1.75)	*p* = 0.064
Internet browsing	13.96	15.71	11.33	15.70	13.78	*p* = 0.102	1.62 (1.85)	1.20 (1.26)	1.25 (1.32)	2.07 (2.18)	2.00 (2.23)	**
Playing e-games	34.36	17.83	14.95	57.12	53.28	***	3.32 (2.95)	1.42 (1.43)	1.64 (1.61)	3.58 (2.80)	4.19 (3.27)	***
Video viewing	64.61	72.83	65.91	62.83	56.52	***	3.05 (2.15)	2.90 (1.86)	2.99 (2.12)	3.04 (2.20)	3.26 (2.42)	*p* = 0.148
Unweighted Obs	3379	976	884	865	654		2944	869	755	765	555	

Note: * *p* < 0.05, ** *p* < 0.01, *** *p* < 0.001

### Are Screen Activities Bad for Adolescent’s Happiness??

**Fig. 1:**
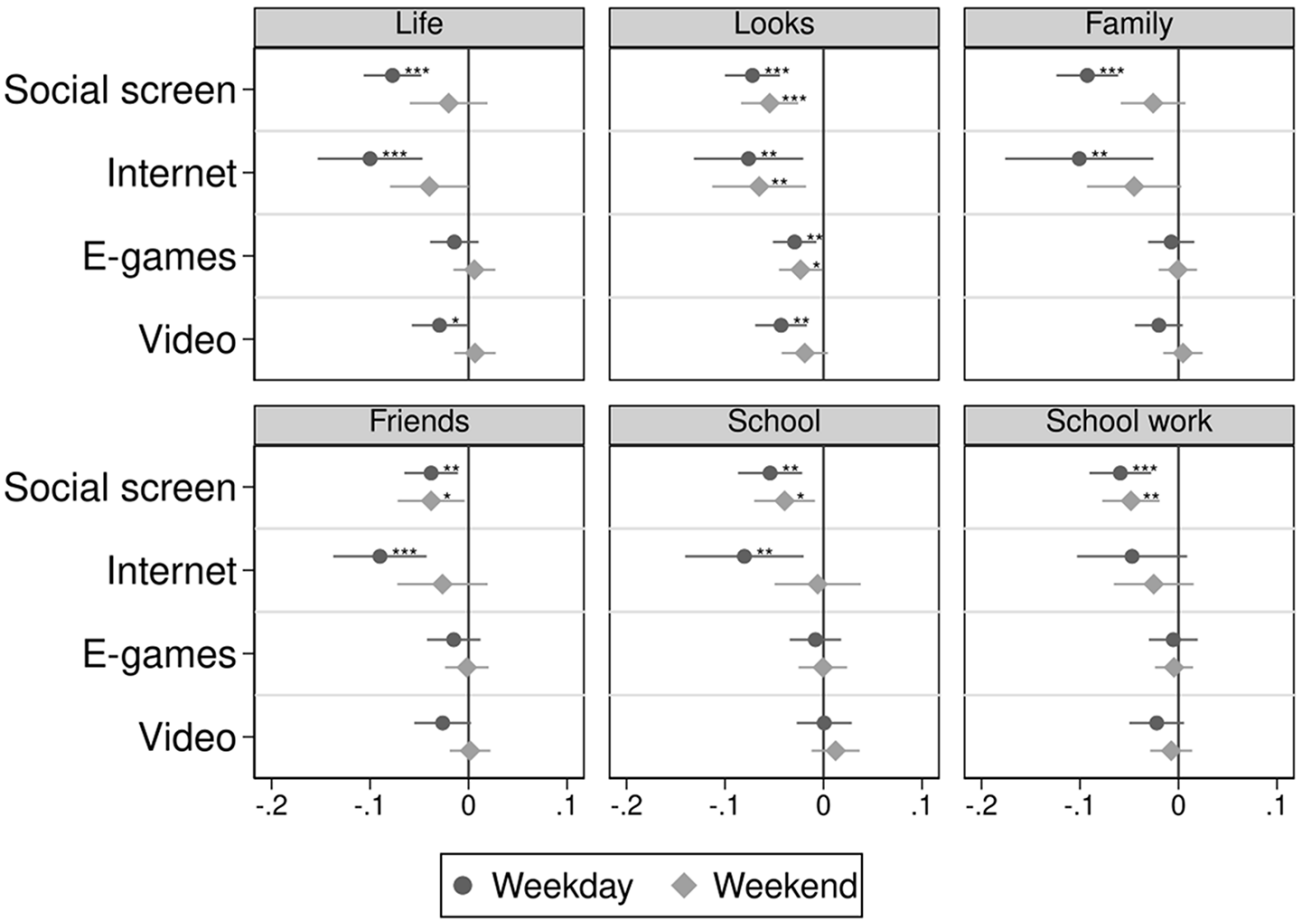
Coefficient plots of screen time on happiness. Note: *p<0.05, **p<0.01, ***p<0.001

Figure 1 below shows the coefficient plots of the regression estimates of an additional hour of screen activity on adolescent happiness, during the weekday and weekend. Since the full time portfolio is considered in this analysis, the tables report the coefficient estimates of β, γ, δ, and ε in model (1) as an additional hour of screen activity contingent on an hour less sleep. Full estimates are shown in Appendix Table A5 and A6.

More screen time, regardless of type, is associated with poorer adolescent’s happiness with their looks (-0.07SD for social screen time and internet browsing, -0.03SD for e-games, and − 0.04SD for watching videos). The p-values from the test for equality in coefficient sizes show that the estimates for social screen time and internet browsing are not significantly different from each other, but are larger than for e-games and video viewing. Given that − 0.07SD is a third of the magnitude of having worse wellbeing as a result of having a long-term illness (-0.22SD), the magnitude of the estimates for social screen time and internet browsing is non-trivial.

More social screen time and internet browsing is associated with lower happiness in all six domains of life, except that internet browsing is not significantly associated with happiness with school work. Besides happiness with looks, more time playing e-games or watching videos is not statistically significantly associated with happiness and magnitudes are close to zero. All of the estimates are larger in magnitude if the activity is performed on a weekday compared to at the weekend.

Estimates where physical activity is omitted is also shown in Appendix Tables A7 and A8, where we may expect that an additional hour of screen time instead of exercise may be detrimental to happiness more than an additional hour of sleep. However, estimates show that omitting physical activity instead of sleep show a similar narrative, where estimates are slightly smaller than if sleep is omitted by about 0.01SD difference on average. It is unclear why this is the case, as we might expect larger estimates for happiness with looks if screen time crowds out physical activity. However, it may be that adolescents’ engagement in physical activity is relatively low to begin with and thus there is not much power in showing the change between an hour of screen time and exercise.

### Is the Relationship between Screen time and Wellbeing Monotonic?

While more screen time may be associated with poorer mental wellbeing, this relationship may not necessarily indicate a more-is-worse relationship i.e., negative and monotonic. Instead, recall the “Goldilocks Hypothesis”, which posits that there may be an inverted U-shape relationship between screen use and mental wellbeing, or that there are negative coefficient estimates for “low” and “high” levels of screen time, and close to zero estimates for “middle” levels of screen time. Very low levels of screen time may indicate deprivation, very high levels of screen time may indicate obsessive use of screen time, and there may be a level of screen use in between that is “just right” and even positively associated with mental wellbeing.

To examine these relationships, I examine the predicted marginal effects of spending 0–12 h on each screen activity, across all happiness domains as shown in Figures A3 and A4. 12 h is used as the cutoff as there were very few adolescents (*n* < 15) who spent more than 12 h on screens. I further re-ran two estimations; (1) squared terms for each screen activity (see Appendix Figure A5 and A6) and (2) dividing hours spent on each screen activity into terciles (see Appendix Table A9 and A10).

Overall, there is little support for the “Goldilocks hypothesis” across all screen activities. The marginal effects for e-games and watching videos only show small negative associations with adolescents’ happiness, and these associations do not become significantly worse at excessive hours (12 h) of screen use. There is a small suggestive positive association between low or mid-levels of passive video viewing and happiness when examining terciles of screen hours. The lowest tercile of passive video viewing on a weekday is positively associated with greater happiness with school work, and the magnitude is significantly different to the top tercile (*p* < 0.000 in testing for equality of coefficients). The middle tercile on a weekend of passive video viewing is positively associated with happiness with life, family, and school work. These suggest some positive relationships from watching videos, possibly educational or videos watched with peers such as family members.

There is a linear, negative and monotonic relationship between social screen time and internet browsing for adolescents’ happiness across all three estimations. This is especially the case for happiness with life and their family during the weekday, and happiness with their looks on the weekend. In particular, much higher levels of screen time (e.g., 6 h compared to 2 h, or the top tercile compared to the lowest tercile) show large negative estimates between happiness with looks, friends, and school in relation to internet browsing. While confidence intervals are large, this is suggestive that there may be particularly detrimental associations to excessive time on the internet. When using squared estimates, there is a small indicative concave relationship between weekend internet browsing and happiness with school, friends and school work, but this points more towards detrimental associations at excessive internet use, with small negative or positive associations at below 6 h of internet use.

### Differences by Gender and Parental Education

**Fig. 2:**
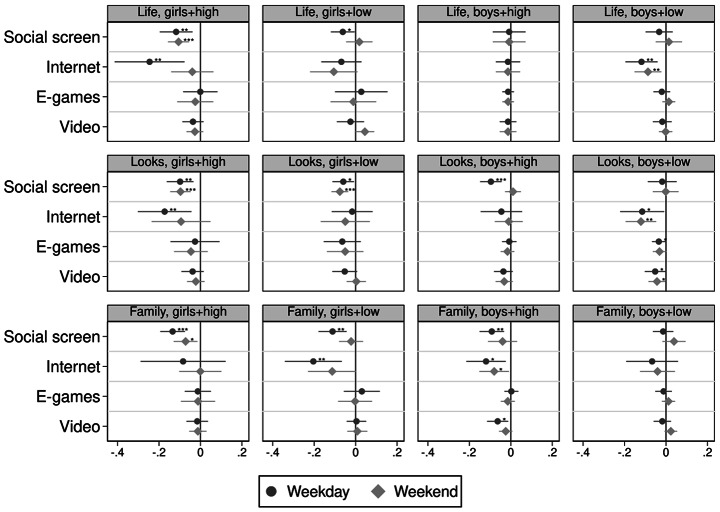
Coefficient plot of interaction results: happiness with life, looks, and family. Note: * *p* < 0.05, ** *p* < 0.01, *** *p* < 0.001

**Fig. 3:**
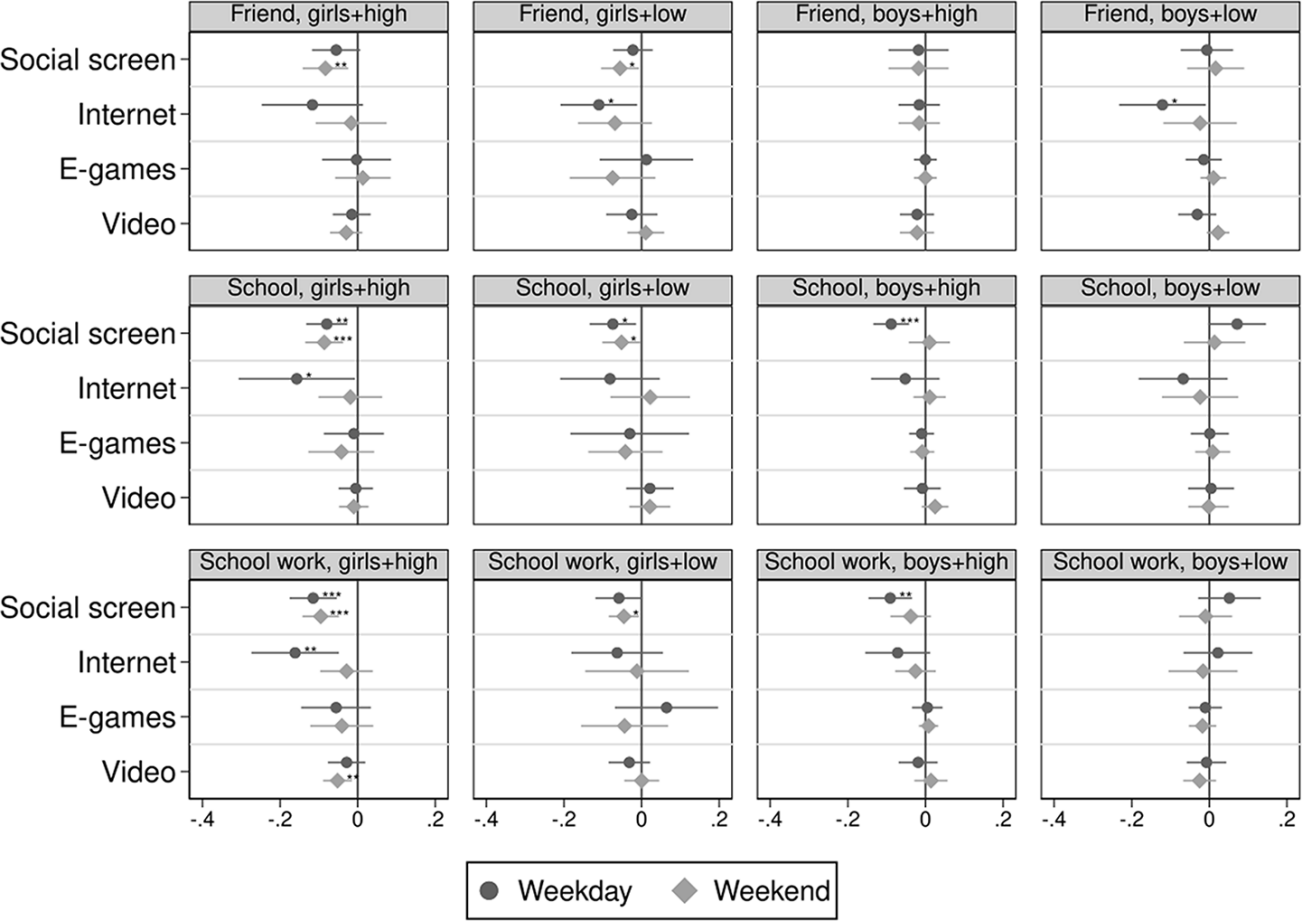
Coefficient plot of interaction results: happiness with friends, school, school work. Note: * *p* < 0.05, ** *p* < 0.01, *** *p* < 0.001

To examine the variations by gender and parental education, I fit model (1) separately for all four groups of adolescents’ gender and parental education. Figure 2 reports the coefficient plots for adolescents’ happiness with their life, looks, and family, while Fig. 3 reports the coefficient plots for adolescents’ happiness with their friends, school, and school work. All coefficient plots report weekday and weekend estimates. Regression tables are reported in Appendix Table A10 and A11.

In the first two columns of Figs. 2 and 3 about girls, an additional hour of social screen time instead of sleep is associated with poorer happiness in all six domains, regardless of parental education. For girls with high parental education, weekday internet browsing is also associated with poorer happiness with their looks (-0.12SD), school (-0.16SD), and school work (-0.16SD), while for girls with lower parental education, weekday internet browsing is associated with lower happiness with family (-0.20SD) and friends (-0.11SD). The magnitude of these estimates across parental education for girls are similar, indicating parental education is not a protective factor for the harmful associations of screen use. Estimates for playing e-games or watching videos are close to zero or statistically insignificant.

Notably, for boys with lower parental education, only more weekday internet browsing is associated with lower happiness with life (-0.12SD), their looks (-0.11SD), and school (-0.12SD), but all other screen activities are not significantly associated with their happiness scores. For boys with high parental education, weekday social screen time is associated with poorer happiness across all domains, and internet browsing is associated with lower happiness with family (-0.12SD).

### Robustness Check

Not all of the adolescents reported both a weekday and weekend time diary. To examine whether there were unobserved differences between adolescents who reported both time diary days and those who did not, I re-run my estimates using a constrained sample of adolescents who only reported both days (*n* = 3,085). As reported in Appendix Table A13, my estimates are robust to the choice of sample, yielding similar estimate sizes and statistical significance.

## Discussion

This paper set out to ask three research questions regarding screen use and adolescent wellbeing using a cohort study of UK adolescents in 2015. First, how do four types of screen use relate to adolescent’s positive wellbeing? My estimates show that more time spent on any screen is adversely associated with happiness with looks. This may be driven by content shown on screen (social comparisons), but also the potential for these activities to displace physical activity and hence poorer confidence with appearance. However, coefficient estimates are larger for social screen time and internet browsing than playing e-games and video viewing, of which the latter two are also statistically insignificant for other domains of happiness. The smaller and statistically insignificant estimates for e-games are in line with few existing studies (Sanders et al., 2019; Przybylski & Weinstein, 2019), suggesting that playing e-games may bring beneficial communication with peers, and may be an effective leisure activity. This may also be the case for video viewing, that is a more ‘normalised’ screen activity, which can be done with family and friends, and more easily monitored by parents.

Second, not all screen activities follow a negative monotonic relationship, only for social screen time and internet browsing, where more time on these activities is associated with worse happiness scores. This provides some support for the displacement hypothesis that these screen activities are crowding out more potentially beneficial activities for adolescents’ wellbeing. The negative estimates are larger in magnitude over the weekday instead of the weekend, suggesting larger substitution with other beneficial activities on a school day. I do not find any curvilinear relationship either, but rather close to zero estimates for playing e-games and video viewing even at high levels of screen activity.

Lastly, the harmful associations between social screen time and happiness are worse for girls than boys, regardless of parental education. The finding is in line with other studies that used the MCS (Banthorpe et al., 2020; Kelly et al., 2019) as well as other studies in the UK (Mcdool et al., 2020; McNamee et al., 2021) that show that girls are more at risk due to harmful content on SNS such as online harassment, poorer body image, and poorer sleep quality.

### Limitations

This paper is limited by not observing content. Therefore, my findings may be showing differences in the way the user engages with screen use. For example, social networking sites which allow users to engage more instantly in small but frequent quantities, compared to browsing the internet where users are consuming other user’s posted content in longer time frames. I cannot discern whether there are harmful content posted on each of these screen platforms.

My findings also only reflect the time use of one cohort of adolescents in the UK, at 2015, and the final sample is self-selected due to the longitudinal nature of the study, and sample selection. Hence, these findings are not generalisable to the wider UK population.

## Conclusions

Relationships may be missed if we do not clarify the types of screen activities we refer to. Being specific helps create more defined policy measures that implicate adolescents’ mental wellbeing. For example in 2021, China banned children and teenagers from online gaming on school days, and limited their time spent on this to one hour a day at the weekend or holiday evenings (Reuters, 2023). If playing games is used by disadvantaged families as a more convenient and accessible way to socialise than an alternative outside paid activity, then restricting gaming time reduces certain families’ options for leisure. Restricting one activity may not necessarily translate into spending more time on productive activities. It is worth noting that weekday estimates are larger in magnitude than weekend estimates, suggesting there are potentially larger and more detrimental substitution effects happening during the weekday. Hence, the recommended 2 h a day by the World Health Organization (Loo et al., 2022) should be abided by for social media or browsing the internet on a school day for adolescents.

A consistent finding is that social screen time is bad for adolescents’ happiness with their looks, especially for girls with higher parental education. This suggests the importance of the role of parents/guardians and teachers in moderating SNS use. A policy recommendation would be to integrate screen use and mental health awareness into the Personal, Social, Health and Economic education curriculum, with a special focus on girls. Letters from schools can help signpost guardians or parents to third party software that help monitor or moderate screen use (e.g., parental controls). As device use and the internet become integral in our daily lives, parents and teachers need to guide adolescents on how to responsibly use these platforms, and encourage offline activities such as sports or games, in line with recommendations of the 24-hour integrated activity guidelines (Lua et al., 2023). This is conditional however, on the level of parental trust on government guidelines.

There is extensive scope for future studies to study the types of content viewed and shared on screens, habits related to screen use such as multi-tasking and content creation, and their relationships with adolescents’ mental wellbeing. As this study is about adolescents in 2015, more needs to be understood on how these mechanisms can be applied or built on after Covid-19 lockdowns, as there are now a generation of adolescents who experienced an intensification of social media and screen use during the lockdowns, likely to affect their behaviours with online platforms today (Hamilton et al., 2023). Finally, more research is required to understand the potential mechanisms as to why socioeconomic status may not always be a protective factor against the harmful associations of screen use.

## Data Availability

The Millennium Cohort Survey data can be accessed online through the UK Data Service for registered users.
